# Health-Based Audible Noise Guidelines Account for Infrasound and Low-Frequency Noise Produced by Wind Turbines

**DOI:** 10.3389/fpubh.2015.00031

**Published:** 2015-02-24

**Authors:** Robert G. Berger, Payam Ashtiani, Christopher A. Ollson, Melissa Whitfield Aslund, Lindsay C. McCallum, Geoff Leventhall, Loren D. Knopper

**Affiliations:** ^1^Intrinsik Health Sciences Inc., Mississauga, ON, Canada; ^2^Aercoustics Engineering Limited, Mississauga, ON, Canada; ^3^Intrinsik Environmental Sciences Inc., Mississauga, ON, Canada; ^4^Department of Physical and Environmental Sciences, University of Toronto, Toronto, ON, Canada; ^5^H.G. Leventhall – Consultancy, Surrey, UK

**Keywords:** wind turbines, infrasound, low-frequency noise, health, human perception, noise, sound pressure level, annoyance

## Abstract

Setbacks for wind turbines have been established in many jurisdictions to address potential health concerns associated with audible noise. However, in recent years, it has been suggested that infrasound (IS) and low-frequency noise (LFN) could be responsible for the onset of adverse health effects self-reported by some individuals living in proximity to wind turbines, even when audible noise limits are met. The purpose of this paper was to investigate whether current audible noise-based guidelines for wind turbines account for the protection of human health, given the levels of IS and LFN typically produced by wind turbines. New field measurements of indoor IS and outdoor LFN at locations between 400 and 900 m from the nearest turbine, which were previously underrepresented in the scientific literature, are reported and put into context with existing published works. Our analysis showed that indoor IS levels were below auditory threshold levels while LFN levels at distances >500 m were similar to background LFN levels. A clear contribution to LFN due to wind turbine operation (i.e., measured with turbines on in comparison to with turbines off) was noted at a distance of 480 m. However, this corresponded to an increase in overall audible sound measures as reported in dB(A), supporting the hypothesis that controlling audible sound produced by normally operating wind turbines will also control for LFN. Overall, the available data from this and other studies suggest that health-based audible noise wind turbine siting guidelines provide an effective means to evaluate, monitor, and protect potential receptors from audible noise as well as IS and LFN.

## Introduction

Wind-based energy production has been identified as a clean and renewable resource that does not produce any known emissions or harmful wastes (1). As a result, wind power has become the fastest growing source of new electric power generation, with several countries achieving high levels of wind power capacity and overall penetration (2). Within the last decade, wind power generation has increased substantially in Canada. In the province of Ontario alone, 1,700 MW (5% of Ontario’s energy generation) have been installed since 2006, with an additional 2,000 MW expected to be installed by the end of 2014 (3). Public support for the use of wind energy is typically high; however, acceptance of projects at the local level does not always reflect this trend. While support is found in some locations, strong opposition stemming from concerns of visual esthetics, health risk perception, and noise levels can be found in others (4–7).

Currently, there exists an ongoing debate surrounding the relationship between wind turbines and human health within both the public and the scientific communities (8). This debate is driven by the fact that some people that live near wind turbines have reported adverse health effects such as (but not limited to) ringing in ears, headaches, lack of concentration, vertigo, and sleep disruption that they attribute to the wind turbines. Some argue that reported health effects are related to wind turbine operational effects [e.g., electromagnetic fields (EMF), shadow flicker from rotor blades, audible noise, low-frequency noise (LFN), and infrasound (IS)]; others suggest that when turbines are sited correctly, reported effects are more likely attributable to a number of subjective variables, including nocebo responses, where the etiology of the self-reported effect is in beliefs and expectations rather than a physiologically harmful entity (9–13). Indeed, there are numerous peer-reviewed studies on the issue and governmental reviews of these studies (14–16).

It is well known that exposure to excessive levels of audible noise, regardless of the source, can cause annoyance, sleep disturbance, cognitive impairment, and other serious health effects. According to the World Health Organization (WHO), nighttime exposure to noise levels above 55 dB(A) outdoors averaged over the year is considered increasingly dangerous for public health and a sizeable proportion of the population will be highly annoyed and sleep-disturbed (17). As a result, jurisdictions across the globe have developed noise regulations specific to wind turbine projects to protect the public from potential noise-related health effects (Table 1). Guidelines are found at various levels of governmental structure including country, state/province, and county/municipality. The list in Table 1 is not globally comprehensive yet is wide-ranging and inclusive of numerous jurisdictions. Though some variability exists among jurisdictions, the majority of the guidelines center around an outdoor limit between 35 and 45 dB(A). This limit coincides with the WHO Europe nighttime noise guideline of 40 dB(A) outdoors, a health-based value derived to “*protect the public, including the most vulnerable groups such as children, the chronically ill and the elderly, from the adverse health effects of night noise*” (17).

**Table 1:** **Current or proposed wind turbine noise limits per jurisdiction**.

Country/region	Noise limits	Reference
Australia/New South Wales	“For a new wind farm development, the predicted equivalent noise level (L eq, 10 min), adjusted for any excessive levels of tonality, amplitude modulation, or low frequency, but including all other normal wind farm characteristics, should not exceed 35 dB(A) or the background noise (L 90) by more than 5 dB(A), whichever is the greater, at all relevant receivers not associated with the wind farm, for wind speed from cut-in to rated power of the WTG and each integer wind speed in between. The noise criteria must be established on the basis of separate daytime (7 a.m. to 10 p.m.) and night-time (10 p.m. to 7 a.m.) periods” … “criteria have been set to restrict noise generated by wind turbines to 5 dB(A) below the lowest acceptable noise criteria for a suburban or rural amenity area [which is 40 dB(A) at night]”	(18)
Australia/South	Background noise is to be measured at the wind farm at various wind speeds at which the turbines operate to determine masking effects of wind generated noise at relevant receiver locations. Noise level predictions are to be identified at all relevant receiver locations. Wind farm noise levels, which may be adjusted for tonality, should not exceed “35 dB(A) at relevant receivers in localities, which are primarily intended for rural living, or 40 dB(A) at relevant receivers in localities in other zones, or the background noise (L Aeq, 10) by more than 5 dB(A), whichever is greater.” Wind turbine setback distances are then based on these criteria	(19)
Australia/Western	Sound generated from wind farms should not exceed 5 dB(A) above the background sound level or 35 dB(A) using a 10-min L Aeq, whichever is greater. Measurements are to be taken at noise-sensitive premises. Setback limits are based on data obtained from sound studies with a 1 km guideline	(20, 21)
Australia/Victoria	Noise level limits are set in accordance with the New Zealand Standard NZS 6808:2010 where “the level of sound from a wind farm should not exceed the background sound level by more than 5 dB, or a level of 40 dB L A90 (10 min), whichever is the greater” … “despite any other condition of this permit, no plans will be endorsed by the responsible authority, and no variation to the endorsed plans will be approved by the responsible authority, which allow a turbine to be located with 2 km of an existing dwelling … unless evidence has been provided to the satisfaction of the responsible authority that the owner of the dwelling has consented in writing to the location of the turbine”	(22, 23)
Canada/Alberta	The minimum basic sound level used to calculate the permissible sound level is 40 dB(A) L eq nighttime with adjustments made for proximity to transportation and population density. The night noise limits should remain between 40 and 56 dB(A) LA eq, based on the number of other residences and existing infrastructure noise sources. For most wind energy locations, the night noise limits will probably fall between 40 and 46 dB(A) LA eq. The day noise limits are 10 dB(A) above night limits	(24)
Canada/British Columbia	Outdoor sound levels measured at an existing residence are to not exceed a maximum of 40 dB(A) based on wind speed 8–11 m/s. More specifically, “where ambient conditions are 35 dB(A) or less: night-time criterion: L eq, 9 h of 40 dB(A) between 10:00 p.m. and 7:00 a.m.; Day-time criterion: L eq, 15 h of 40 dB(A) between 7:00 a.m. and 10:00 p.m.; Ambient conditions are to be assumed at 35 dB(A) for calculation purposes. Where ambient conditions are shown to be >35 dB(A) during either the day or night (except where another wind power project is present), a 5 dB(A) increment may be applied to a measured background sound level to determine the day or night criterion, to a maximum of 50 dB(A)”	(25)
Canada/Manitoba	Sound limits are based on the levels recommended by CanWEA where a sliding scale based on wind speed is used. These levels start at 40 dB(A) at a wind speed of 4 m/s and rise to 53 dB(A) at 11 m/s. For setback limits, sound modeling-based assessments have been used to determine that 500–550 m from a receptor (an occupied dwelling) is sufficient to ensure that the sound criteria can be met	(26)
Canada/New Brunswick	At a wind speed of 7 m/s the overall noise limit is 40 dB(A), this value increases with increasing wind speeds to 53 dB(A) at speeds ≥10 m/s. Proposed wind farms must demonstrate compliancy with these guidelines for all sensitive receptors, including homes and recreational areas within 1 km of the turbine. These values are used to determined setback distances	(21, 27)
Canada/Ontario	“If the wind turbine(s) are audible in a recording (does not include extraneous noise sources) then additional analysis is required for the subject recording: determine the value of the 10 min L eq via software or obtain it directly from the recording device; determine if the wind turbine noise is tonal; obtain the average wind speed at the microphone height (1.5 or 4.5 m) over the 10 min recording session.” “Results of the10 min L eq (including tonal penalty if applicable) are to be compared against the applicable sound level limits contained in the 2008 Noise Guidelines” where at standardized wind speeds at 10 m height from below 5–10 m/s the sound level limit ranges from 40 to 51 dB(A)	(28)
Canada/Quebec	Based on a review by the Minnesota Department of Commerce, municipalities determine setbacks in the Province of Quebec, with 500 m being the most commonly used setback distance. No noise guidelines were reported. However, it does appear the Province of Quebec has a nighttime rural noise limit (zone 1) of 40 dB(A) that is not wind turbine specific	(21)
Denmark	“The total noise impact from wind turbines may not exceed the following limit values: (1) at the most noise-exposed point in outdoor living area no more than 15 m from dwellings in open countryside: (a) 44 dB(A) at a wind speed of 8 m/s. (b) 42 dB(A) at a wind speed of 6 m/s. (2) At the most noise-exposed point in areas with noise-sensitive land use: (a) 39 dB(A) at a wind speed of 8 m/s. (b) 37 dB(A) at a wind speed of 6 m/s”	(29)
Germany	“For immission points outside buildings, the binding immission values for the rating level are (a) in industrial areas 70 dB(A); (b) in commercial zones during the day 65 dB(A) at night 50 dB(A); (c) in core areas, village areas, and mixed-use zones during the day 60 dB(A) at night 45 dB(A); (d) in general residential areas and small residential estate areas during the day 55 dB(A) at night 40 dB(A); (e) in purely residential areas during the day 50 dB(A) at night 35 dB(A); (f) in spa areas, for hospitals and nursing homes during the day 45 dB(A) at night 35 dB(A)”	(30)
Ireland	A minimum setback distance of 500 m has been suggested, but is not absolute “because of the lack of correlation between separation distance and wind turbine sound levels, the use of a defined setback of turbines … is not appropriate” …. An outdoor limit of 40 dB(A) “attributed to one or more wind turbines, should be applied in order to restrict noise from wind turbines at noise sensitive properties” was defined. Post construction noise levels can be measured at wind farms to confirm if noise regulations are being met	(31)
New Zealand	“The level of sound from a wind farm should not exceed the background sound level by more than 5 dB, or a level of 40 dB L A90 (10 min), whichever is the greater. About 40 dB is typical of a quiet residential area with only light traffic and natural sounds such as the wind in the trees. In contrast, sound levels along-side an urban road would be around 60–70 dB during the day and about 50–60 dB at night. There are some locations that are particularly quiet at times and so the recommended limit of 40 dB would be considered to be unreasonable” …. “Where a local authority has identified in its district plan the need to provide a higher degree of protection of acoustic amenity. The standard recommends that when particular conditions are met, the sound from the wind farm during the evening and night time should not exceed the background sound level by more than 5 dB or a level of 35 dB LA90(10 min), whichever is the greater”	(23)
UK/England	For both day and night time, noise is recommended to be limited to 5 dB(A) above background noise. There is a fixed night limit of 43 dB(A) using L A90 (10 min) or 45 dB(A) for properties benefiting financially from wind turbine development. A penalty of up to 5 dB(A) may be added if a distinct tone is distinguishable. England has no minimum setback distance though the noise limits suggest a minimum of 350 m for a typical wind turbine	(32)
USA/Oregon	For noise generated by a wind energy facility, the assumed background L50 noise levels if 26 dB(A) or the actual ambient background level. “The noise levels from a wind energy facility may increase the ambient statistical noise levels L10 and L50 by more than 10 dB(A)” …. Noise levels at the appropriate measurement point are predicted assuming that all of the proposed wind facility’s turbines are operating between cut-in speed and the wind speed corresponding to the maximum sound power level established by IEC 61400-11 (version 2002-12)	(33)
USA/Massachusetts	Massachusetts has draft “Promising Practices for Nighttime Sound Pressure Levels by Land Use Type” for wind turbine noise. These values were provided in a 2012 report (Wind Turbine Health Impact Study). MassDEP convened a technical advisory group to consider potential revisions to its noise regulations and policy. The promising practices for nighttime sound pressure levels are industrial areas 70 dB(A); Commercial areas 50 dB(A); villages, mixed usage 45 dB(A); sparsely populated areas, 8 m/s wind 44 dB(A); sparsely populated areas, 6 m/s wind 42 dB(A); residential areas, 8 m/s wind 39 dB(A); residential areas, 6 m/s wind 37 dB(A). Wind speeds should be measured at 10 m above ground, outside of residence, or location of concern	(14)
USA/New Hampshire	No noise limit has been imposed by the State. However, the State Site Evaluation Committee (SEC) has accepted a 45 dB(A) setback on at least one project (e.g., Groton Wind Project; Groton, New Hampshire)	(34)
USA/Maine	The State of Maine has Sound Level Limits for Routine Operation of Wind Energy Developments in Chapter 375 of Rule Chapters for the Department of Environmental Protection. The sound levels resulting from routine operation of a wind energy development shall not exceed (a) 75 dB(A) at any time of day at any property line of the wind energy development or contiguous property owned or controlled by the wind energy developer, whichever is farther from the proposed wind energy development’s regulated sound sources; and (b) 55 dB(A) between 7:00 a.m. and 7:00 p.m. (the “daytime limit”), and 42 dB(A) between 7:00 p.m. and 7:00 a.m. (the “nighttime limit”) at any protected location	(35)

Even when these health-based noise limits are met, some people living near wind turbines self-report a variety of adverse health effects that they attribute to living near the wind turbines (8, 16). As a result, the etiology of these health effects has been hypothesized by some to stem from exposure to low-frequency sounds, including IS (0.01–20 Hz) and LFN (10–200 Hz) (36–38), both of which are known components of the broad-band sound associated with normal wind turbine operation (9, 39, 40). For example, in 2011 Møller and Pedersen (38) stated “*Even when A-weighted levels are considered, a substantial part of the noise is at low frequencies, and for several of the investigated large turbines, the one-third-octave band with the highest level is at or below 250 Hz. It is thus beyond any doubt that the low-frequency part of the spectrum plays an important role in the noise* ….” In response to these concerns, a number of investigations (published since 2010) have measured IS and LFN associated with modern wind turbine operation at a variety of distances, operating scenarios, and geographic and meteorological conditions (Tables 2 and 3). Collectively, these reports suggest that sound associated with well-functioning wind turbines has measurable energy within the IS and LFN spectra. However, IS levels, which are often described in dB(G), are consistently well below auditory perceptual levels (41–45) and LFN is below available guidelines (42). Furthermore, IS levels at relatively close distances to wind turbines are equivalent to or less than those produced by a number of natural or engineered sources that individuals are exposed to on a regular basis (43, 44, 46). The physical characteristics of sounds emitted from wind turbines have been recognized to influence the perception and annoyance to wind turbine associated sounds; however, this generally refers to sounds that are above the auditory level of perception (10, 47, 48).

**Table 2:** **Review of reported wind turbine IS emissions (reported after 2010^a^)**.

Author	Reference	WT rated power	Distance (m)	IS	Overall sound level	Background sound level	Wind speed (m/s)
Ambrose et al.	(51)	1.65	520	51–64 dB(G) indoor	18–24 dB(A) indoor	39–44 dB(G) indoor	6–20
				54–65 dB(G) outdoor	41–46 dB(A) outdoor	49–54 dB(G) outdoor	

Boczar et al.	(41)	2	131	55–70 dB SPL outdoor	Not reported	Not reported	1–8

Turnbull et al.	(43)	2.1	85	72 dB(G) outdoor	Not reported	Not reported	6–8
			185	67 dB(G) outdoor	
			360	61 dB(G) outdoor	
		2	100	66 dB(G) outdoor	Not reported	62 dB(G) outdoor	
			200	63 dB(G) outdoor	

Evans et al.	(44)	2.1	1500	49–56 dB(G) indoor	Not reported	51–55 dB(G) indoor	10–12
				57–61 dB(G) outdoor	Not reported	58–60 dB(G) outdoor	
		2.1	1400	57–66 dB(G) indoor	Not reported	Not reported	
				56–62 dB(G) outdoor	Not reported	Not reported	

Evans	(45)	3	1800	40–70 dB(G) indoor	Not reported	45–60 dB(G)	1–18
			2700	45–70 dB(G) indoor	Not reported	45–70 dB(G)	1–22

*^a^In addition to the studies cited here, others have measured wind turbine associated IS; however, only those that explicitly reported ranges were included in this table*.

**Table 3:** **Review of reported wind turbine LFN emissions (reported after 2010^a^)**.

Author	Reference	WT rated power	Distance (m)	LFN	Overall sound level	Background sound level	Wind speed (m/s)
O’Neal et al.	(42)	2.3	305	63.5 dB(C) outdoor	49.4 dB(A) outdoor	Nor reported	3.3
			323	54.7 dB(C) indoor	33.8 dB(A) indoor		3.2
		1.5	290	47.1 dB(C) indoor	27.1 dB(A) indoor	Not reported	6.2
			305	62.8 dB(C) outdoor	50.7 dB(A) outdoor		3.3
			312	50.6 dB(C) indoor	33.6 dB(A) indoor		6.4

Evans et al.	(46)	2.1	1500	0–4 dB(A) indoor	NA	3–8 dB(A) indoor	10–12
				21–25 dB(A) outdoor		22–29 dB(A) outdoor	

*^a^In addition to the studies cited here, others have measured wind turbine associated LFN; however, only those that explicitly reported ranges were included in this table*.

It has been suggested that wind turbine noise limits set in dB(A), which simulates the sensitivity of human hearing and perception, may underestimate the contribution of IS and LFN from wind turbines (37). Alternative sound weightings, including G-weighting [dB(G)] and C-weighting [dB(C)], have been proposed as more appropriate metrics for noise limits when LFN and IS are present, respectively (37, 49). However, Health Canada recently suggested that, in the case of wind turbine noise, there was “*no additional benefit in assessing LFN as C- and A-weighted levels were so highly correlated (r = 0.94) that they essentially provided the same information*” (50). Accordingly, the purpose of this paper is to examine further IS, LFN, and overall sound levels typically produced by wind turbines and provide discussion as to whether concerns regarding wind turbine associated IS and LFN are warranted. Field measurements of outdoor LFN and overall sound levels and indoor IS at locations between 400 and 900 m from the nearest wind turbine, which were previously underrepresented in the scientific literature, are reported. The results of these measurements are put into context with existing published works and current available guidelines based on dB(A) to provide a weight-of-evidence conclusion.

## Materials and Methods

### Indoor infrasound measurements

Sound measurements were conducted in three residences, two at 450 m and one at 900 m from the nearest wind turbine. These turbines were part of an operating wind farm with over 40 turbines, each with a power capacity of 1.5 MW. The measurements were carried out using Class 1 instrumentation with sufficiently low-frequency range and noise floor. Measurements were carried out on a ground plane fitted with a double windscreen. The double wind screen consisted of the thin hemispherical wireframe (450 mm diameter) covered with a thin layer (approximately 10 mm) of open cell foam. This setup is consistent with that defined in IEC 61400-11 with the exception that the measurement location was at a dwelling rather than close to a wind turbine. Although not in a windy environment, a double windscreen helps protect very low frequency and infrasonic measured levels against pressure fluctuations within a dwelling caused by moving air from ventilation and opening/closing doors.

For these measurements, access was not available to turbines in order to conduct on/off tests for quantifying ambient levels. Additionally, turbine power performance was not made available during the study. In order to identify whether the turbines in the facility were operating, an autocorrelation technique was used in the signal analysis in order to detect characteristics in the sound signal attributable to the turbine operation. This autocorrelation technique (52) exploits the periodicity in the signal attributable to the wind turbine operation and uses this feature to detect when the turbines were operating. IS levels measured during wind turbine operation were compared to those when the wind turbines were unlikely to be operational (i.e., at wind speeds below turbine cut-in at 3 m/s). Data were collected from 1 to 1000 Hz and subsequently weighted using dB(G) to focus the analysis on the IS component, and allow for comparison to other studies.

The data presented in this report represent the periods where 1-min interval recordings showed the existence of the wind turbine noise (i.e., characteristic blade passage frequencies) the clearest out of the entire measurement period, which was 3–4 weeks. Because the nature of the signal detection mechanism, and the averaging over a minute, the Type A uncertainty for the measured value is difficult to quantify. The Type B uncertainty of the measurement is that of a Class 1 instrumentation, which is typically ±1 dB.

### Outdoor low-frequency noise and overall sound measurements

Sound levels were measured near two different wind turbine facilities, both with more than 30 wind turbines each. The turbines had a power capacity between 1.5 and 2.4 MW. Measurements were carried out outdoors at 4.5 m height, and at a distance between 400 and 800 m. Meteorological data were also recorded at a height of 10 m at the same location. The sound measurements were carried out using Class 1 instrumentation with sufficiently low noise floor. A large 450 mm diameter spherical secondary windscreen was employed in addition to a commercially available 7 cm primary wind screen to minimize pseudo-noise from wind flowing over the microphone. Field sound measurements of wind turbines are highly susceptible to contamination from extraneous noise such as from human activity, fauna, insects, and wind-induced noise. To control for these sources of contamination, the following methods were used:

- sound measurements were only collected during nighttime, between 10 p.m. and 5 a.m.;
- measurements were conducted in 1 min intervals;
- measurements were binned by wind speed for each 1 min interval;
- intervals within 1 h of rainfall or snowfall were not used; and
- intervals with gusty winds (>2 m/s above the mean wind speeds) were not used as these periods are more susceptible to wind-induced pseudo-noise.

Measurements were carried out in the vicinity of the wind facility during wind turbine operation as well as with the turbines off. The same filtering and data quality management methods were applied to both data sets. A minimum of 60 data points in each wind bin were gathered. To isolate only the LFN portion of the spectrum, data between 20 and 200 Hz were analyzed and summed. Once tallied, the mean spectrum for the 3 and 6 m/s integer wind speeds was calculated. For each of those cases, the calculation was made from spectra where the mean wind speeds were within 0.5 m/s of the stated value and was relatively steady during the entire interval. The gust filtering ensures that no gust was more than 2 m/s above the mean. The mean spectrum was calculated by computing the energy averaged sound level for each 1/3rd octave band between 20 and 200 Hz, and then computing an A-weighted sum of the spectrum.

The self-noise emitted by the system itself was assessed using the measurements conducted during periods when the wind turbines in the vicinity were not operating. The mean spectrum at various wind speeds was compared to those found in other literature comparing measured ambient levels with respect to wind speed. The most applicable study, conducted by the Japanese Ministry of Environment and reported by Tachibana (53) compared sound levels measured with various windscreens ranging from naked microphone to a specialized dodecahedron double windscreen. Measured low-frequency levels were at or below those reported in the double windscreen case in the Japanese study for most wind speeds and locations. It should be noted that although the measured ambient levels are consistent with those measured with high degree of windscreen protection, pseudo-noise contamination of the signal cannot be fully avoided.

Based on the measurements conducted, the typical measured SD for the A-weighted level was ±3 dB for the turbines ON, and ±2 dB when the turbines were OFF. The SD was higher at lower wind speeds and decreased with increasing wind speed. This is due to wind-induced ambient noise (which is fairly steady) dominating the signal at the higher wind speed. At lower wind speeds, because the ambient levels are lower, individual non-turbine related events such as vehicular traffic, faunal noise, or other intermittent noises increase the variability in background noise. Additionally, during lower wind speeds, the wind turbine noise source would be more susceptible to changes in wind speed at the hub. For example, for two cases where the ground level wind speed is 3 m/s, the hub height wind speed could be 4 m/s in one case and 8 m/s in another. This would result in a difference in the amount of noise produced by the turbine. It is the authors’ view that given the above variability, wind turbine noise measurements at far field distances should carry a nominal uncertainty value of ±3 to ±5 dB.

## Results and Discussion

### Indoor infrasound measurements

Infrasound levels in the homes at 450 m were relatively similar, measuring 59 and 58 dB(G) (Table 4). IS measured at the furthest location of 900 m was comparable to the measurements at 450 m, measuring 60 dB(G). These data indicate that IS levels were relatively constant with increased distance from the nearest wind turbine and were approximately 25 dB below the level of human perception [approximately 95 dB(G) (54)], which may be indicative of non-wind turbine associated distant sources of IS. The results reported here are consistent with previous measurements at varying distances (41–45). For instance, IS measurements from 290 to 323 m from wind turbines were 20–30 dB below the human auditory threshold levels (42). Additional measurements of IS in the 1–30 Hz range at a distance of 200 m from the wind turbines also remained below the human auditory threshold (41). Other investigations have shown that at further distances (1.5 km) indoor IS levels in two residences were between 49 and 61 dB(G), with no reported difference between operational and shutdown periods, also suggesting that there are other sources of IS contributing to these results (44). The same group (55) also showed that indoor IS levels were between 50 and 70 dB(G) at distances of 1.8 and 2.7 km from the nearest wind farm. In conjunction with these reports, the results from the current field investigation indicate that wind turbines are a source of IS; however, sound levels are well below the human auditory threshold.

**Table 4:** **Indoor infrasound measured at three homes at two different distances to 1.5 MW turbines**.

WT rated power (MW)	Distance (m)	IS level [dB(G)]
1.5	450	59
1.5	450	58
1.5	900	60

Only two jurisdictions have developed clear guidelines for IS and neither is specific to wind turbine noise (Table 5). This may be partly a result of the highly sophisticated equipment and analyses required to accurately measure IS and distinguish between the IS generated from wind turbines and other natural and engineered sources (56). The Queensland Department of Environment and Resource Management’s Draft *ECOACCESS Guideline-Assessment of Low Frequency Noise* proposed an interior IS limit of 85 dB(G) (57). This value was derived based on a 10 dB protection level from the average 95 dB(G) hearing threshold (54) and previous Danish recommendations for IS limits (58). The Japanese Handbook on Low Frequency Noise provides an IS reference value of 92 dB(G) at 10 Hz and 1/3-octave bands up to 80 Hz (59). These values were derived from investigations that monitored complaints of mental and physical discomfort from healthy adults exposed to low-frequency sounds in a room (59). Though the Japanese guidelines were derived through short-term monitoring experiments and are not equivalent to the long-term exposure associated with living in proximity of wind turbines, the levels of IS measured as part of this current study (Table 4) are 20–30 dB below these guidelines.

**Table 5:** **Infrasound noise limits per jurisdiction (not wind turbine specific)**.

Country/region	Noise limits	Reference
Australia/Queensland	G-weighting function used to determine annoyance due to infrasound within the frequency range from 1 to 20 Hz. The recommended limit value for infrasound inside dwellings during the day, evening and night is 85 dB(G). Noise is measured over a 10-min period and a 5 dB penalty is added for impulsive noise. Approximate determination of sound pressure level may be made by analysis of the signal using one-third octave bands and application of the provided weighting values	(57)
Japan	The reference value for complaints of mental and physical discomfort include the G-weighted sound pressure level of 92 dB(G) as measured at 10 Hz	(59)

A limited number of reports have suggested that the IS component of wind turbine noise is the cause of self-reported adverse health effects (51, 60, 61). Mechanisms within the inner ear that are sensitive to low levels of IS stimulation have been proposed to be associated with adverse health responses (36, 37, 62). However, functional magnetic resonance imaging has provided powerful evidence that IS is perceived via similar auditory pathways as audible sounds when above the level of perception with no indication of cortical activation at sub-threshold values (63). Furthermore, exposure to IS is known to originate from other engineered or natural processes, including wind and weather systems (64), volcanic (65) and auroral activity (66), and mountain ranges (67); this would arguably also induce stimulation of the inner ear. Recent outdoor measurements have provided an indication of IS levels from a number of natural sources, including sea waves at 25 m from the coast [75 dB(G)], 250 m from a coastal cliff face [69 dB(G)] and 8 km inland from the coast [57 dB(G)] (43). The authors reported that wind turbine IS levels, which were between 61 and 72 dB(G) at distances of 85–360 m, were lower than many of the natural sources measured (43). IS is also generated in urban environments as a result of human activity and engineered sources such as industrial processes, ventilation systems, and vehicles (43, 44). Measurements of IS in a typical urban setting have been reported to be up to 70 dB(G) during the daytime and 63 dB(G) at night (44). In comparison, studies reporting biological responses to IS exposure were at sound pressure levels that were above the level of auditory perception, much higher than those produced by wind turbines [e.g., 145 dB and 165 dB (68, 69)]. Collectively, these reports and the measurements from the current investigation indicate that humans are regularly exposed to IS from several natural and engineered sources at levels that exceed those produced by wind turbines. Although sounds with impulsive characteristics (e.g., wind turbines) generate greater levels of annoyance than non-impulsive sources, annoyance levels have only been associated with noises that are above the threshold of auditory perception (9, 70). Our measurements of IS, and those from the literature, are all well below the threshold of auditory perception.

### Outdoor low-frequency noise and overall sound measures

**Figure 1:**
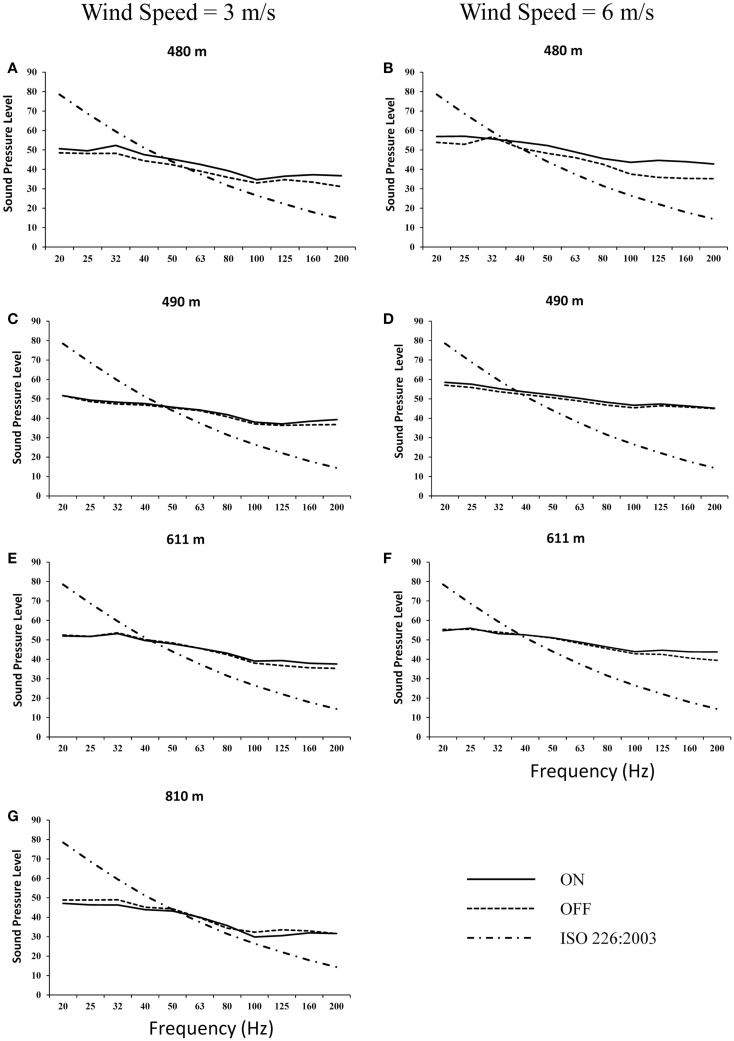
**Outdoor low-frequency noise measurements at 480 m (A,B), 490 m (C,D), 611 m (E,F), and 810 m (G) from 1.5 MW wind turbines with wind speeds of 3 m/s (A,C,E,G) and 6 m/s (B,D,F) with turbines on and off. Hearing threshold (ISO 226:2003) is also provided**.

Outdoor LFN levels were assessed through 1/3-octave band measurements with wind turbines operational (on) and during scheduled shutdown periods (off) at distances of 480, 490, 611, and 810 m (Figure 1). The most evident result is the similarity between measured LFN levels with wind turbines on and off (ambient) from 20 to 100 Hz, at which point sound levels began to deviate from one another. This deviation was most apparent at measurements taken nearest the turbines (Figures 1A,B) where levels of LFN from turbines on differed from ambient by 6 and 9 dB at wind speeds of 3 and 6 m/s, respectively. As distance from the turbines increased, the amount by which LFN levels measured with turbines on and off differed compared those observed at 480 m. At 490 and 611 m, the maximum difference between on and off was between 3 and 4 dB. At the furthest observation point of 810 m (3 m/s), there was no difference in LFN levels measured during wind turbine operation and shutdown (Figure 1G). At lower frequencies within the LFN spectrum (20–100 Hz), the contribution of the wind turbines was negligible when compared to ambient levels at distances ≥490 m (Figures 1C–G). At all distances and wind speeds, irrespective of wind turbine operation, LFN exceeded the ISO-defined audible threshold at frequencies >40–50 Hz (71). These results indicate that the observed increase in LFN during wind turbine operation was found primarily in the frequency range consistent with the audible range of hearing, namely 20–20,000 Hz, and not in the IS range (<20 Hz). It is also noted that the same applies to ambient noise levels, namely that the levels cross the auditory threshold at frequencies between 40 and 50 Hz and higher.

**Figure 2:**
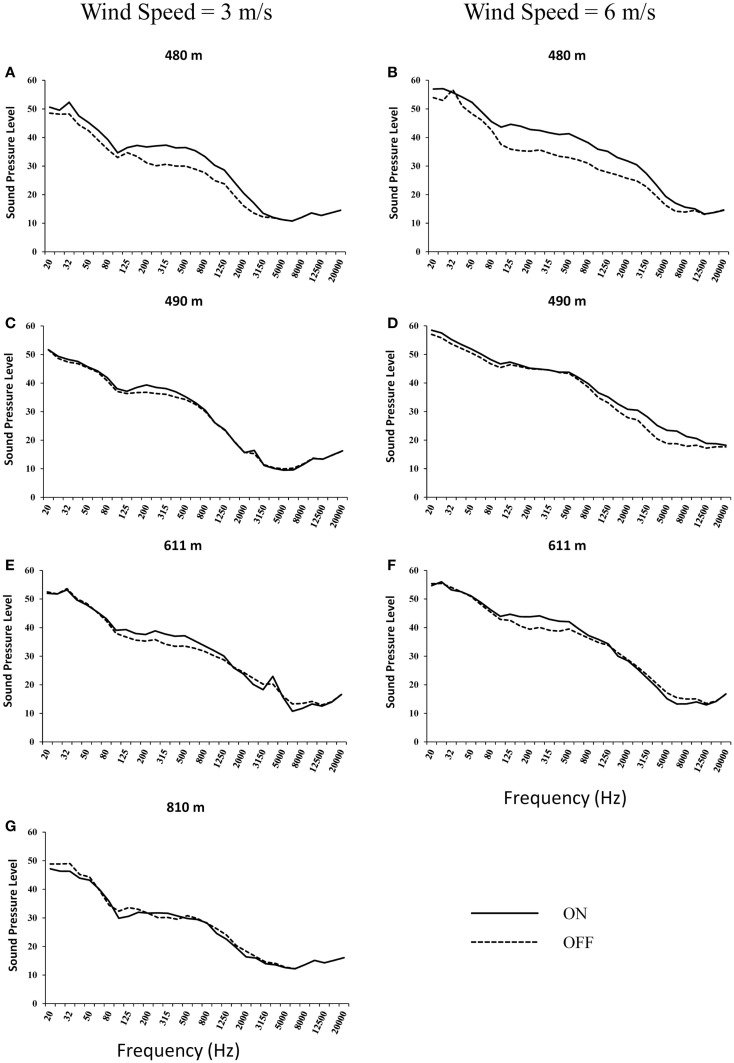
**Outdoor sound measurements at 480 m (A,B), 490 m (C,D), 611 m (E,F), and 810 m (G) from 1.5 MW wind turbines with wind speeds of 3 m/s (A,C,E,G) and 6 m/s (B,D,F) with turbines on and off**.

Through the 1/3-octave band analysis of overall sound levels (20–20,000 Hz; Figure 2), it was apparent that the increase in LFN from wind turbine operation was accompanied by increased sound levels at higher frequencies (i.e., >200 Hz). This was particularly evident at 480 m where wind turbine associated sound levels continued to be above ambient levels until approximately 3150 Hz (Figures 2A,B). At further distances, sound levels were above ambient levels at frequencies between 125 and 1000 Hz, but not easily distinguishable from ambient levels below 125 Hz or above 1 kHz (Figures 2C–F). These results indicate that though there was an observed increase in LFN levels during wind turbine operation at the 480 m location this increase was accompanied by an increase in sound levels up to 3 kHz.

At closer distances where the LFN component can be measured above the ambient conditions, the mid frequency sound levels were also above ambient levels. In those cases, the signal-to-noise ratio of the mid frequency sound levels was higher than that below 125 Hz, indicating that the most audible portion of the frequency spectrum was between 125 and 3150 Hz. At further distances, it was evident that the signal-to-noise ratio decreased, such that only acoustic energy between 125 Hz and about 800–1000 Hz was above background, with the highest signal-to-noise ratio between 200 and 500 Hz (Figures 2C–F). The single measurement point at 810 m showed no measurable increase in any of the mean sound levels. This is indicative that a presence of LFN in the signal from wind turbines was accompanied by a presence in mid frequency sound levels. For instance, where the LFN levels were considerably above ambient levels, the mid frequency sounds levels were also considerably increased. This indicates that, at the distances of interest, it is the mid frequency region that is the most audible portion of the noise from the turbines. Only at closer distances, where the mid frequency components would be clearly audible (6–9 dB signal-to-noise ratio), would the low-frequency components from the turbines start to be audible above ambient levels. The overall A-weighted sound pressure level was significantly affected by the mid frequency component. As a result, it would be expected that by controlling the overall sound pressure level [dB(A)] from normal functioning wind turbines that the LFN component would also mitigated.

When the wind turbines were operating, the highest mean LFN level [dB(A)] was observed at 480 m (Table 6). At the other locations >480 m from the wind turbines, the measured difference between wind turbines on and off was between 1 and 3 dB, at least half of that observed at 480 m. The mean overall sound levels reported in dB(A) showed very similar trends to those reported in the LFN analysis. Critically, the increase in mean sound levels at the closest location (480 m) reported in the LFN spectrum and overall sound in the 1/3-octave band analysis was maintained. In addition, the observed trends at 490, 611, and 810 m, also remained consistent. From these results, it is evident that during wind turbine operation, the increased sound levels that began in the LFN spectrum, at approximately 160 Hz and continued to 1000 Hz, were above auditory threshold levels and represented in the mean dB(A) sound measures. The consistency between the mean dB(A) measurements and trends observed in the 1/3-octave band analysis suggest that the contribution of the LFN component and overall sound levels were accounted for in the calculation.

**Table 6:** **Low-frequency noise (LFN) and overall sound levels with turbines on and off (i.e., background) in dB(A)**.

Wind speed (m/s)	Distance (m)	LFN “On”	LFN “Off”	Overall sound “On”	Overall sound “Off”^a^
3	480	30	26	41	35
	490	32	30	40	39
	611	31	30	42	40
	810	25	26	36	36
6	480	36	30	47	40
	490	39	38	49	48
	611	37	34	49	45

*^a^Ambient noise at this location, with turbines off, is influenced by wind speed (3 and 6 m/s) and movement of vegetation in the measuring location*.

A number of investigations have reported LFN levels in the proximity of wind turbines (Table 2) similar to those reported here. Furthermore, the results showing LFN levels passing the auditory threshold between 40 and 50 Hz are similar to those that have been previously reported (42, 72). For instance, O’Neal et al. (42) measured indoor and outdoor LFN levels from wind turbines at a distance of 300 m and found the levels were below the United Kingdom’s (UK) Department for Environmental and Rural Affairs (DEFRA) and Japanese guidelines and became audible at approximately 50 Hz (42). Elsewhere, LFN levels were only marginally higher and remained well below guidelines even though measurements were taken as close as 104 m from the nearest wind turbine (72). LFN measured at 1.8 and 2.7 km from the nearest wind farm was comparable during pre-operational and operational periods of development, though small increases at frequencies above 63 Hz were reported (45). At a greater distance of 1.5 km from wind turbines, Evans et al. found LFN levels were similar to those measured at distances of 10 and 30 km from the turbines (46). Further, organized shut downs of the two wind farms showed that the contributions of the turbines to LFN measurements were negligible or relatively small contributions at 100 Hz and above (46). As shown with IS, LFN is also produced by natural and common engineered sources: in urban environments, including offices and residences, LFN levels often exceed available guidelines and are greater than those measured 1.5 km from the nearest wind turbine (46).

The sound characteristics and associated fall off with distance have been extensively measured by Tachibana in the range from 0.8 Hz to 5 kHz at 164 locations around 29 wind farms, using one third octave analysis. The average of the measures fell with a slope of 4 dB/octave over the whole range. The average passed through 55 dB at 10 Hz and crossed the hearing threshold at about 50 Hz (73). Other, less detailed measurements on individual turbines have shown slopes of 5 dB/octave to 6 dB/octave (42). A spectrum, which falls at 5 dB/octave and passes through, for example, 60 dB at 10 Hz has an A-weighted level of 39 dB(A), which is mainly determined by a broad peak in the A-weighted spectrum in the region of 200 Hz to 630 Hz. Any shift in the level at 10 Hz is reflected in the A-weighted level. Similarly, this spectrum has a C-weighted level of 58 dB(C). The difference between dB(A) and dB(C) levels depends only on the spectrum shape and is independent of overall level, indicating that for similar spectrum shapes, the dB(A) and dB(C) levels are highly correlated.

There are currently no widely accepted international health-based limits for LFN specifically derived for wind turbines. A number of jurisdictions have developed both indoor and outdoor LFN limits to address potential issues associated with industrial noise emissions (Tables 7 and 8). The majority of the limits are for indoors and utilize 1/3-octave sound pressure level measurements between 5 and 200 Hz. This analysis enables assessors to identify tonal components within the spectrum that may be problematic. The 1/3-octave band limits vary significantly between jurisdictions. In Poland, LFN limits are around 10 dB(A) across 1/3-octave bands between 10 and 250 Hz (74). In Denmark, LFN is limited to a total level of 20 dB(A) between 10 and 160 Hz (75), while in UK, guidelines are generally between 10 and 25 dB(A) depending on the frequency between 10 and 100 Hz (76). Indoor LFN limits provide a basis to address specific complaints from local residents; however, for wind farm development, regular monitoring of outdoor sound levels presents a more practical option.

**Table 7:** **Indoor LFN noise limits per jurisdiction (not wind turbine specific)**.

Country/region	Noise limits	Reference
Australia/Queensland	Overall sound pressure level inside residences should not exceed 50 dB (linear). If the dB (linear) measurement exceeds the dB(A) measurement by more than 15 dB further analysis of one-third octave band between 20 and 200 Hz is suggested. Recommended limits for non-tonal low-frequency noise in a dwelling, during the evening and night is 20 dB(A) and during the day 25 dB(A)	(57)
Denmark	Low-frequency noise limits are limited to a total level of 20 dB(A) indoors as measured by the A-weighted level of noise in 1/3-octave bands between 10 and 160 Hz	(75)
Japan	Reference values for complaints of mental and physical discomfort are provided in 1/3-octave sound pressure levels from 10 to 80 Hz. The handbook suggests taking sound pressure level and G-weighted sound pressure levels. The guidelines provided by the handbook are only applicable to LFN produced by stationary sound sources that produced LFN continuously and is not applicable to LFN from transient and intermittent sources such as airplanes, railways, or explosive blasts. Values for mental and physical complaints were based on an investigation of tolerable levels of low-frequency noise from which a 10 percentile curve was developed	(59)
Poland	“Criteria were based on the measurement data of annoying noises, investigation of the effects of noise on the health of the exposed inhabitants, laboratory tests of thresholds of narrow and broad-band noise perception and a review of the present literature. In order to assess the noise spectra measured in dwellings, the A10 characteristic has been accepted as the rating curve. Its levels, L, for 1/3-octave bands are determine by the relation La10 = 10-Ka, where Ka is the A-weighting. Low-frequency noise is annoying when the sound pressure levels of the noise exceed the A10 curve and simultaneously exceed the background noise level by more than 10 dB for tonal noise and by 6 dB for broad-band noise”	(74)
United Kingdom	Indoor recordings of Leq, L10 and L90 in third octave bands between 10 and 160 Hz should be made. If the Leq exceeds values provided then it may indicate a significant source of LFN that could be causing disturbances. If the noise only occurs during the day then a 5 dB relaxation may be applied. If the noise is steady then a 5 dB relaxation may be applied. Reference curve was developed based on protective value of 5 dB below the average threshold of hearing	(76)

**Table 8:** **Outdoor LFN noise limits per jurisdiction (not wind turbine specific)**.

Country/region	Noise limits	Reference
Australia/New South Wales	Considered it unnecessary to establish the full spectral signature of all wind turbines based on the findings that wind turbines have very similar spectral signatures and do not generate excessive levels of low-frequency noise. Recommended using dB(C) measurements at intermediate locations to identify any anomalies such as a mechanical problem or a need for any further investigation. “Trigger levels of 65/60 dB(C) as suggested by Broner (2011) were adopted” “5 dB(A) penalty should be applied to the predicted or measured noise level from the wind farm for the periods and meteorological conditions under which the low-frequency noise has been identified.” *New South Wales Industrial Noise Policy (1999)* suggests that a difference of 15 dB or greater between dB(A) and dB(C) weightings can establish the presence of a low-frequency noise can be established and addressed	(77)
Australia/South Australia	Follow the suggestions made by the New South Wales Industrial Noise Policy, but do not provide any specific limit or required actions	(19)
Canada/Alberta	A LFN issue exists both when “(A) the time-weighted dB(C)–dB(A) value for the measured daytime or nighttime period is ≥20 dB and (B) A clear tonal component exists at a frequency between 20 and 250 Hz.” When a LFN issue has been identified, measurements of C- and A-weighted scales are to be made concurrently. The presence of a LFN issue is confirmed when both “(A) The isolated time-weighted average dB(C)–dB(A) value for the measured daytime or nighttime period is ≥20 dB. For the 1/3-octave frequency bands between 20 and 250 Hz and below: (a) the linear sound level of one band must be at least 10 dB or more above one of the adjacent bands within two one-third octave bandwidths (b) there must be at least a five dB drop in level within two bandwidths on the opposite side of the frequency band exhibiting the high sound levels.” If these conditions exist, “5 dB(A) must be added to the measured comprehensive sound level. If this value exceeds the permissible sound level, the licensee must identify the source of the LFN and implement noise attenuation measures to address the issue in a timely way”	(24)
Japan	Reference values for outdoor measurements of low-frequency noise to provide guidance in how to address complaints of rattling windows and doors are provided for 1/3-octave bands from 5 Hz up to 50 Hz as reported in L eq. The values were based on rattling thresholds observed in two studies. At 5 Hz, the maximum value is 70 dB L eq and increases up to 99 dB L eq at 50 Hz	(59)

Only a small number of jurisdictions, including the province of Alberta, Canada (24), Japan (59), and Australian States of South Australia and New South Wales (18), have introduced outdoor LFN noise limits (Table 8). Several of these guidelines determine the difference between C- and A-weighted sound measurements (19, 24, 77). This calculation can provide an indication of an unbalanced spectrum; a difference >20 dB between two weightings may warrant further investigation based on those regulations (78, 79). The ability of this calculation to predict LFN issues is limited, particularly when there are low levels of background noise that result in a large difference between the A- and C-weighted sound levels that are not associated with increased levels of annoyance (80). In the current investigation the difference between wind turbine operational scenarios (i.e., on and off) was <5 dB at the 490 and 611 m locations at both wind speeds. Measured background levels at 490 and 611 m were also high, measuring 48 and 45 dB(A), respectively. A number of noise guidelines, including those in UK (32), New Zealand (23), and several of the Australian states (18–20, 22), take into account the potential for high levels of background noise by suggesting that the contribution of wind turbines to be limited to <5 dB above background. In the current investigation, the 480 m location was the only one observed to be ≥5 dB above background levels (6 dB at 3 m/s and 7 dB at 6 m/s).

## Conclusion

Data from the current investigation indicate that wind turbines produce noise that is broad-band in nature, which includes energy within the IS and LFN spectrums. Based on the data presented here, the indoor IS component of wind turbine noise measured as dB(G) at distances of 450 and 900 m, was well below the levels of human perception (54), providing further support to previous reports (39, 41–45, 81). IS is produced at levels comparable or greater than those shown here by natural and engineered sources (43, 81). There is no scientific evidence to indicate that exposure at these G-weighted levels of IS can directly impact human health. Recent studies have indicated that psychological factors (12, 13) and the manner in which information is presented from media reports and non-scientific sources may influence the perception and expectations associated with wind turbine sounds (82). These reports suggest that subjective variables may be a more likely etiology for self-reported effects than from exposure to IS associated with normal wind turbine operation.

The LFN analysis showed that when the turbines were both on and off sounds above 40–50 Hz exceeded the threshold for auditory perception as defined by ISO 226:2003 (71). A clear contribution from the operation of the wind turbines was only observed at the closest location of 480 m when compared to background levels. Increases in LFN observed between 100 and 200 Hz corresponded to increases in overall sound measures reported in dB(A). The use of alternative sound weightings [i.e., dB(C)] may have utility in instances where there are significant increased levels of LFN, particularly when a tonal component is present. However, the results from the current investigation indicate that increases in LFN associated with wind turbine operation are correlated with increases in overall sound levels. These results, in conjunction with those of previous reports, suggest that controlling for overall sound levels produced by normally operating wind turbines will inherently control for LFN (38, 48, 77). The results reported here are in agreement with a recent report issued by Health Canada, which concluded that following over 4,000 h of wind turbine noise measurements, there was “*no additional benefit in assessing LFN as C- and A-weighted levels were so highly correlated (r = 0.94) that they essentially provided the same information*” (50). Given the low levels of IS and the correlation between LFN and overall sound levels from wind turbines, the development and enforcement of suitable outdoor guidelines and limits, based on dB(A), provide an effective means to evaluate, monitor, and protect potential receptors.

## Author Contributions

All authors contributed equally to the conception and design of the analysis; PA performed the field measurements and analyzed the data; RB wrote the manuscript with contributions from all other authors.

## Conflict of Interest Statement

In terms of competing interests (financial and non-financial) the authors work for various consulting firms and have worked with wind power companies. The authors are actively working in the field of wind turbines as environmental health scientists and acoustical engineers. Although we make this disclosure, we wish to reiterate that as independent scientific professionals our views and research are not influenced by these contractual obligations.
